# Air-Liquid Interface System To Understand Epstein-Barr Virus-Associated Nasopharyngeal Carcinoma

**DOI:** 10.1128/mSphere.00350-18

**Published:** 2018-07-18

**Authors:** Sharon E. Hopcraft, Cary A. Moody, Blossom Damania

**Affiliations:** a Department of Microbiology and Immunology, University of North Carolina at Chapel Hill, Chapel Hill, North Carolina, USA; b Lineberger Comprehensive Cancer Center, University of North Carolina at Chapel Hill, Chapel Hill, North Carolina, USA

**Keywords:** ALI, EBV

## Abstract

Epstein-Barr virus (EBV) infects epithelial cells and is associated with epithelial malignancies. Although EBV reactivation is induced by epithelial differentiation, the available methods for differentiation are not widely used. In a recent study, Caves et al. (mSphere 3:e00152-18, 2018, https://doi.org/10.1128/mSphere.00152-18) explored the use of a new transwell-based air-liquid interface (ALI) system to differentiate EBV-infected nasopharyngeal carcinoma cells. They found that cells cultured in the ALI system expressed markers of differentiation and supported complete EBV reactivation. This system offers an easy method for differentiation that could be widely adopted. This system could be extended to other epithelial cell types.

## Abstract (precis)

Epstein-Barr virus (EBV) infects epithelial cells and is associated with epithelial malignancies. Although EBV reactivation is induced by epithelial differentiation, the available methods for differentiation are not widely used.

## COMMENTARY

Epstein-Barr virus (EBV) is a ubiquitous gammaherpesvirus that establishes lifelong infections and has latent and lytic stages of the viral life cycle (1). EBV infects B cells and epithelial cells and is associated with several lymphomas and epithelial malignancies, including gastric cancer and nasopharyngeal carcinoma (NPC) (1). While B cells maintain the latent reservoir of EBV, epithelial cells in the oral mucosa are likely a major compartment for viral replication, as EBV is transmitted through saliva (2–4). However, the study of EBV replication in epithelial cells has been hampered by the lack of robust *in vitro* model systems. NPC lines lose EBV genomes during cell line establishment (5, 6), and most epithelial cells are difficult to infect. Furthermore, in monolayers of some epithelial cell types, induction of the lytic cycle with histone deacetylase or protein kinase C inhibitors can be abortive (7) and may not reflect authentic reactivation pathways *in vivo*. Recent advances have been made in studying EBV infection of epithelial cells with the use of the three-dimensional organotypic raft culture system, which faithfully recapitulates epithelial differentiation. This system involves seeding cells on a feeder cell-containing collagen plug that is lifted to an air-liquid interface (ALI) to induce differentiation. Differentiated cells can be efficiently infected with EBV via the apical surface, and virus disseminates throughout the raft (8). Additionally, cells that have been latently infected with EBV can be differentiated, which induces viral reactivation (9). Thus, differentiation of epithelial cells is critical for the study of EBV in this cell type.

As the organotypic raft culture system is not widely used, Caves et al. explored the use of a transwell-based ALI culture system to probe EBV replication in differentiated epithelial cells (10). The ALI system is conventionally used to polarize primary airway epithelial cells. In this system, cells are seeded on permeable transwell membranes that are thinly coated with collagen and are exposed to air by the removal of media from the apical chamber. If effective, the ALI method could be easily adopted across the field. Caves et al. used ALI to culture HK1 cells, an NPC line in which EBV genomes could not be detected after cell line establishment but which was subsequently latently infected *in vitro* (11). They showed that ALI cultures of HK1 NPC cells express two markers of differentiation, keratin 10 and involucrin, and support complete reactivation of EBV as demonstrated by expression of all viral genes, genome replication, and assembly of virions. It is somewhat surprising that markers of differentiation were induced in these cultures as transformed cells generally lose the ability to differentiate. In fact, C666-1 cells, representing another NPC line that maintained latent EBV infection during cell line establishment, were not capable of forming an intact epithelium and could not be cultured in the ALI system. As NPC is classified into several subtypes based on differentiation (12), perhaps the success of HK1 versus other NPC lines depends on the NPC subtype from which the line was derived. Although EBV is mostly latent in NPC, reactivation of latent EBV is thought to contribute to the carcinogenesis of NPC (13). Thus, this method allows the study of EBV replication and pathogenesis in HK1 NPC cells.

The degree to which these ALI cultures recapitulate epithelial differentiation remains unclear. The use of histology and immunohistochemistry could reveal whether or not these cultures form a stratified epithelium with distinct layers. Furthermore, how closely the ALI system mimics the organotypic raft culture system could be assessed by examining whether ALI cultures are infectible with EBV through the apical surface. Ultimately, ALI cultures may serve as a reasonable complement to the organotypic raft culture system, which is considered the gold standard in epithelial differentiation models.
